# Is single port laparoscopic cholecystectomy superior to standard cholecystectomy in post-operative pain?

**DOI:** 10.1016/j.amsu.2021.01.071

**Published:** 2021-01-30

**Authors:** Talal Mohammed Ali Alshahri, Sabry Abounozha, Rashid Ibrahim

**Affiliations:** a Alfaisal Hospital- Riyadh, Saudi Arabia; b Northumbria Healthcare NHS Foundation Trust, UK; c University Hospitals Plymouth NHS Trust, UK

**Keywords:** Single port cholecystectomy, Post-operative pain, Minimal invasive, Postoperative pain, Advantage of single port laparoscopy, Standard cholecystectomy

## Abstract

A best evidence topic has been constructed using a described protocol. The three-part question addressed was: is single port laparoscopic cholecystectomy superior to standard cholecystectomy in post-operative pain? Using the reported search, 8083 papers were found. 8 studies were deemed to be suitable to answer the question. The outcomes assessed were post-operative pain differ in single or standard laparoscopic cholecystectomy, all study used VAS (visual analogue scale). The evidence showed no difference in post-operative pain for patients went for single laparoscopic in compared with standard laparoscopic cholecystectomy.

## Highlights

• No difference in post-operative pain for patients who had single laparoscopic compared with standard laparoscopic cholecystectomy.
• May differ in hospital stay and cosmesis but not for post-operative pain.
• If your main concern is post-operative pain evidence not supporting any technique over other.

## 1 Introduction

A Best Evidence Topic was constructed based on a structured protocol. This is described by the International Journal of Surgery.

**Fig. 1:**
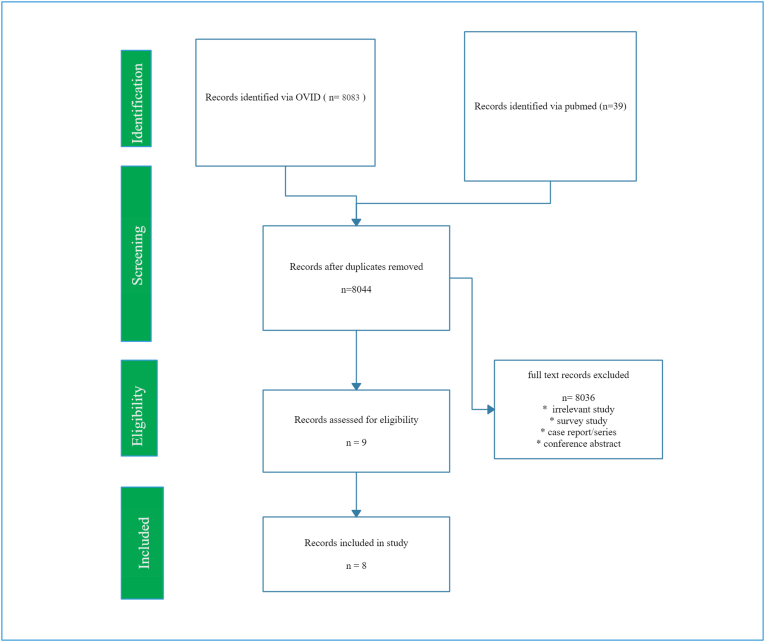
Comparison of post-operative pain among patients who underwent single port laparoscopic cholecystectomy or standard laparoscopic cholecystectomy techniques.

## 2 Clinical scenario

As general surgeon concerning regarding post-operative pain for patients going to laparoscopic cholecystectomy, should we go with singe port rather than standard lap chole with advance of surgical technique?

## 3 Three-part question

In (a patient undergoing a cholecystectomy) is (single port laparoscopic cholecystectomy) superior to (standard laparoscopic cholecystectomy in post op pain)?

## 4 Search strategy

A. Medline using the PubMed interface:

[single port laparoscopic cholecystectomy OR single incision laparoscopic cholecystectomy] AND [standard port laparoscopic cholecystectomy OR conventional laparoscopic cholecystectomy OR multiport laparoscopic cholecystectomy] AND [ postoperative pain OR post-operative pain].

B. Embase 1974 to October 2020 using the OVID interface:

[single port laparoscopic cholecystectomy OR single incision laparoscopic cholecystectomy] AND [standard port laparoscopic cholecystectomy OR conventional laparoscopic cholecystectomy OR multiport laparoscopic cholecystectomy] AND [ postoperative pain OR post-operative pain].

The results were limited to English articles and human studies.

## 5 Search outcome

A total of 8083 papers were found using OVID and 39 using the PubMed interface. A total of 8044 papers were identified after we removed duplicates. Out of these 8044 papers were excluded because they were irrelevant based on titles and abstracts. 8036 full-text articles were screened and assessed for eligibility. From these, 8 papers were identified that provided the best evidence to answer the question eligible articles were defined as those articles that compared the post-operative pain among patients who underwent single port laparoscopic cholecystectomy or standard laparoscopic cholecystectomy techniques, please refer to fig. 1.

## 6 Result

(please refer to the Table 1).

**Table 1:** Structured protocol.

Author, date of publication, journal and country	Study type and level of evidence	Patient group	Outcomes	Key results	Additional comments	Follow up period
Phillips et al. [1], 2011, Surg Endosc, USA	randomized controlled trial, level II	The study included 197 were randomized to SILC (n = 117) or 4PLC (n = 80).	Their aim to compared single port with four port cholecystectomy with the goal of assessing safety, feasibility, and factors predicting outcomes.	single incision laparoscopic cholecystectomy is associated with increased early postoperative pain, (worst pain = 6.3) P = 0.914 (average pain = 4.8) P = 0.350) in single incision group compared to (worst pain = 6.2) P = 0.914 (average pain = 4.5) P = 0.350 in 4 ports laparoscopic cholecystectomy group.	multicenter, single-blind trial, small sample size, advantages they exclude obese, pregnant lady, there were several limitations to this study. First, exclusion in this trial of patients with acute cholecystitis and those who required intraoperative cholangiography makes it difficult to apply the results of this study to all patients undergoing cholecystectomy, second, despite randomization, the SILC group was noted to have lower BMI (28.9 vs. 31.0, p = 0.011) which may limit the application of these results to patients with higher body weights.	12 month
Wong et al. [2], 2012, Surg Laparosc Endosc Percutan Tech, China	Cohort study, level III	20 consecutives patients who underwent SILC (SILC group) were compared with a prospective cohort of 20 patients who underwent conventional 4-port laparoscopic cholecystectomy (LC group) during the same period.	Comparing Postoperative Pain Between Single-incision Laparoscopic Cholecystectomy and Conventional Laparoscopic Cholecystectomy	SILC(VAS = 2.9 ± 1.6) result in significantly less postoperative pain than conventional laparoscopic cholecystectomy (vas = 4.8 ± 1.5), (P < 0.01).	small sample size, prospective confounding factors all of their patients diagnosed with same disease, their limitation, they did short follow up.	one month
Pan et al. [3], 2013, World Journal Gastroenterol, China	randomized controlled trial, level II	one hundred and two patients with symptomatic benign gallbladder diseases were randomized to SILC (n = 49) or TPLC (n = 53).	post-operative pain score between two groups.	Single incision laparoscopic cholecystectomy (VAS = 2.0 ± 1.5) was superior in terms of postoperative pain to triple ports (VAS = 3.5 ± 1.6) with P-value 0.0000).	Single center, prospective randomized control trial, small sample size, advantages they did good Inclusion criteria and Exclusion criteria, limitation: they didn't give clear evidence for underling cause of pain variations.	Two month
Bingener et al. [4], 2015, J Am Coll Surg, USA	double-blinded randomized controlled trial, level II	The study included 110 cases divided in two groups, single port and four ports.	Comparing pain scope on postoperative patients for laparoscopic cholecystectomy between two groups .	Overall pain scores were similar between the Groups p = 0.056.	small sample size, confounding factors were noted they used multiple pain assessment scores, no obvious limitations in this study.	More than one year
Arezzo et al. [5], 2016, Surg Endosc, Italy	randomized controlled trial, level II	A total of 600 patients were randomly assigned to receive either SPC (n = 297) or MPC (n = 303) and were eligible for data analysis.	Comparing Score of Postoperative pain in both groups.	There were no significant differences in pain between two groups.	large sample size, Multicentric, RCT, confounding factors were noted pain recorded daily for the first week and then weekly up to 60 days after surgery, there were several limitations to this study that must be discussed. First, approximately 25% of patients were lost to follow-up at 1 year, which is higher than would be normally expected for a 12-month prospective study. Second, all surgeons participating in this trial had performed at least 15 previous SPC cases	60 days
Tyagi et al. [6], 2017, journal of minimal access surgery, India	randomized controlled trial, level II	Seventy-five patients were included in the SLC arm and 75 in the SILC arm between September 2012 and 2014.	post-operative pain scores in comparing between two groups.	No significant difference was found in duration and intensity of pain between two procedures p > 0.05	Single center, randomized control trial, small sample size, advantages they did great randomization to the two groups using the sealed envelope technique which was opened just before the skin incision, their limitations in excluding criteria for obese and pregnant lady.	24 h
Casaccia et al. [7], 2019, Journal of the Society of Laparoscopic & Robotic Surgeons, Italy.	Cohort study, level III	The study included 40 patients for laparoscopic cholecystectomies comparing single ports with multiple ports during October 2016 till October 2017.	Difference in two groups in term of pain post laparoscopic cholecystectomy.	In their study, according to visual analogue scale evaluation, the pain profile was similar but SPLC group was associated with more analgesic's requirement.	Single center, small sample size, disadvantage of this study were short period and small sample size.	Not determined
Klein et al. [8], 2020, Langenbeck's Archives of Surgery, Germany.	randomized controlled trial, level II	The study included 193 patients between December 2009 and June 2011 to compare between single-incision laparoscopic cholecystectomy and multiport laparoscopic cholecystectomy.	The primary endpoint was postoperative pain on the first day after surgery.	pain was similar between the two groups on the morning of the first postoperative day (p = 0.021).	small sample size, Single center, confounding factors were dividing pain to, pain in first day and pain on discharge, the comparative data were collected prospectively, limitation in the comparability of techniques is the use of different follow-up periods and considerable heterogeneity in results and methodologies between the individual studies.	mean of 70.4 months

## 7 Discussion

Phillips et al. conducted a small randomized controlled trial multicenter, single-blind in 2011 they included 197 patients who underwent laparoscopic cholecystectomy. (117) 59.3% of the laparoscopic cholecystectomy were single incision and 40.6% (80) were multiple. The author concluded that single incision laparoscopic cholecystectomy is associated with increased early postoperative pain and was shown to be an independent variable in worst and average pain scores, (6.3–4.8) (worst-average pain) (P = 0.914–0.350) in single incision group compared to (6.2–4.5) (worst-average pain) (P = 0.914–0.350) in 4 ports laparoscopic cholecystectomy group [1].

In contrast, Wong et al., in 2012, conducted a small sample size, From August 2009 to July 2010, 20 consecutives patients who underwent SILC (SILC group) were compared with a prospective cohort of 20 patients who underwent conventional 4-port laparoscopic cholecystectomy (LC group) during the same period. The author concluded that single incision laparoscopic cholecystectomy resulted in significantly less postoperative pain than conventional laparoscopic cholecystectomy (P < 0.01) (2.9 ± 1.6) (4.8 ± 1.5) [2].

Furthermore, pan et al., in 2013 reached single incision laparoscopic cholecystectomy was superior in postoperative pain after they conducted a Single center, prospective randomized control trial which included 102 who randomized to single incision laparoscopic cholecystectomy (n = 49) 48% and triple port laparoscopic cholecystectomy (n = 53) 51.9%. The author noticed postoperative pain in single incision group was (2.0 ± 1.5) and triple ports (3.5 ± 1.6) with P-value 0.0000) [3].

Nevertheless, despite these contradicting findings, another four randomized control trials and one cohort study showed no statically significant difference in post-operative pain between single post laparoscopic cholecystectomy and standard laparoscopic cholecystectomy.

Those are the study which were conducted by Bingener et al. [4] In 2015, which was a randomized controlled trial that included 110 patients who had symptomatic cholelithiasis underwent laparoscopic cholecystectomy, large Multicentric randomized controlled trial in 2016 by Arezzo et al. [5] which included 600 patients who underwent laparoscopic cholecystectomy, on 2017 Tyagi et al. did Single center, randomized control trial that included 75 patients who had symptomatic cholelithiasis underwent laparoscopic cholecystectomy [6], and also casaccia et al., 2019 [7] did single center small cohort study that included 40 patients who had symptomatic cholelithiasis underwent laparoscopic cholecystectomy recently in 2020 klein et al. [8]. Performed a single center retrospective randomized controlled study including 193 patients who underwent laparoscopic cholecystectomy, our message from this review single port laparoscopic cholecystectomy comparing to standard multiple port laparoscopic cholecystectomy no difference in post-operative pain.

## 8 Clinical bottom line

In minimal invasive surgery especially in laparoscopic cholecystectomy single port may differ in hospital stay and cosmesis but not for post-operative pain so our advice if your main concern is post-operative pain evidence not supporting any technique over other.

## Ethical approval

Not applicable.

## Sources of funding

None.

## Author contribution

TA: conducted the literature search and wrote the paper.

SA: assisted in writing the paper.

RI: assisted in the literature search, editing and Writing the paper.

## Declaration of competing interest

None.
